# Meteorological Register

**Published:** 1814-09

**Authors:** C. Blunt

**Affiliations:** Philosophical Instrument Maker, No. 38, Tavistock-Street, Covent-Garden


### METEOROLOGICAL REGISTER.

*From July the 25th, to August the 25th,* 1814.

Kept by C. BLUNT, Philosophical Instrument Maker, No. 38, Tavistock-Street, Covent-Garden.

| Moon. | Day. | Wind. | Barometrical Pressure. Max. | Min. | Mean. | Temperature. Max. | Min. | Mean. | |
|---|---|---|---|---|---|---|---|---|---|
| | 26 | SW | 29·90 | 29·78 | 29 847 | 82 | 59 | 68 6 | Fair |
| | 27 | SW | 29 97 | 29 95 | 29·965 | 83 | 65 | 72·3 | Fair |
| | 28 | SW | 29·90 | 29·75 | 29 79 | 94 | 65 | 78 | Fair |
| | 29 | SW | 29·85 | 29 79 | 29 82 | 85 | 58 | 71 3 | Fair |
| | 30 | SW | 30·05 | 29·91 | 29·995 | 75 | 57 | 65·6 | Fair |
| | 31 | SW | 30·— | 29·94 | 29 97 | 84 | 54 | 68 | Fair |
| ○ | 1 | SbyW | 29·90 | 29·89 | 29 893 | 84 | 56 | 68 3 | Fair |
| | 2 | SW | 30·02 | 30·— | 30·015 | 83 | 57 | 68·6 | Fair |
| | 3 | SSW | 29·98 | 29·94 | 29·957 | 85 | 54 | 68·6 | Fair |
| | 4 | WSW | 30 08 | 30·03 | 30·063 | 77 | 55 | 66·6 | Fair |
| | 5 | W | 29·84 | 29·75 | 29·775 | 76 | 54 | 66·6 | Fair, Showery |
| | 6 | WbyN | 29·83 | 29 73 | 29 776 | 76 | 56 | 65 6 | Fair, Cloudy |
| | 7 | WbyN | 29·80 | 29·72 | 29 76 | 77 | 57 | 66·3 | Fair, Cloudy, Show. du Night |
| ◑ | 8 | W | 29 70 | 29·69 | 29·695 | 71 | 51 | 62·6 | Fair, Cloudy |
| | 9 | NW | 30·— | 29·80 | 29·93 | 72 | 53 | 61·3 | Fair, Clear |
| | 10 | NW | 30·— | 30·— | 30·— | 72 | 54 | 62·6 | Fair |
| | 11 | W | 30·08 | 30·03 | 30·057 | 74 | 56 | 64·3 | Fair |
| | 12 | W | 30·08 | 30·03 | 30·045 | 76 | 55 | 65·3 | Fair |
| | 13 | W | 29·95 | 29·86 | 29·903 | 79 | 56 | 67·3 | Fair |
| | 14 | NW | 29·86 | 29·86 | 29 86 | 80 | 57 | 68 | Showery |
| ● | 15 | NW | 29·89 | 29·86 | 29·892 | 81 | 59 | 69·3 | Fair |
| | 16 | W | 29·84 | 29·79 | 29·802 | 65 | 58 | 61·6 | Fair |
| | 17 | W | 29·95 | 29·90 | 29·932 | 68 | 56 | 61·3 | Fair |
| | 18 | W | 30·— | 29·97 | 29·992 | 66 | 57 | 61·3 | Showery |
| | 19 | NW | 30·— | 29·94 | 29·965 | 70 | 56 | 62·6 | Fair |
| | 20 | NW | 29·97 | 29·95 | 29·985 | 68 | 55 | 62 | Fair |
| | 21 | N | 29·95 | 29·80 | 29·875 | 70 | 57 | 63 6 | Showery |
| ◐ | 22 | W | 29·83 | 29 83 | 29·83 | 71 | 55 | 62 | Showery |
| | 23 | SbyN | 29 70 | 29·64 | 29·67 | 72 | 55 | 62 | Fair |
| | 24 | NE | 29·64 | 29·56 | 29·84 | 74 | 56 | 63 | Rain all Night |
| | 25 | W | 29 60 | 29·57 | 29·589 | 71 | 56 | 62 | Showery |

RESULTS.

| | | | | | |
|---|---|---|---|---|---|
| Mean barometrical pressure | 29·887 | | Mean temperature | 62·85 deg. | |
| Maximum 30·08 | wind at | W | Maximum 94, | wind at | SW |
| Minimum 29·56 | ——— | NE | Minimum 51, | ——— | W |

*Scale exhibiting the prevailing Winds during the Month.*

| N | NE | E | SE | S | SW | W | NW |
|---|---|---|---|---|---|---|---|
| 1 | 1 | 0 | 0 | 1 | 9 | 11 | 8 |

| | Mean barometrical pressure. | Mean temperature. |
|---|---|---|
| From the full moon on the 1st August, to the last quarter on the 8th | 29·891 | 67·2 |
| ——— last quarter, to the new moon on the 15th | 27·355 | 64 48 |
| ——— new moon, to the first quarter on the 22d | 29·920 | 63·1 |

MONTHLY

